# Alpha-Amylase Inhibits Cell Proliferation and Glucose Uptake in Human Neuroblastoma Cell Lines

**DOI:** 10.1155/2022/4271358

**Published:** 2022-07-25

**Authors:** Kateryna Pierzynowska, Sofia Thomasson, Stina Oredsson

**Affiliations:** ^1^Department of Biology, Lund University, Sölvegatan 35, 22362 Lund, Sweden; ^2^Department of Animal Physiology, The Kielanowski Institute of Animal Nutrition and Physiology Polish Academy of Sciences, Instytucka 3, 05110 Jabłonna, Poland; ^3^SGPlus-Group, Alfågelgränden 24, 23132 Trelleborg, Sweden

## Abstract

The present article describes a study of the effects of alpha-amylase (*α*-amylase) on the human neuroblastoma (NB) cell lines SH-SY5Y, IMR-32, and LA-N-1. NB is the most common malignancy diagnosed in infants younger than 12 months. Some clinical observations revealed an inverse association between the risk of NB development and breastfeeding. *α*-Amylase which is present in breast milk was shown to have anticancer properties already in the beginning of the 20th century. Data presented here show that pancreatic *α*-amylase inhibits cell proliferation and has a direct impact on glucose uptake in the human NB cell lines. Our results point out the importance of further research which could elucidate the *α*-amylase mode of action and justify the presence of this enzyme in breast milk as a possible inhibitor of NB development. *α*-Amylase can be thus recognized as a potential safe and natural mild/host anticancer agent minimizing chemotherapy-related toxicity in the treatment of NB.

## 1. Introduction

Neuroblastoma (NB) is the most common extracranial paediatric tumor, and it is the most common malignancy diagnosed in infants younger than 12 months. The incidence rate of NB is almost twice that of leukaemia (the second neonatal cancer) in the mentioned paediatric group [1–4]. Regarding clinical presentation and prognosis, NB demonstrates a variety of patterns, from spontaneous regression to aggressive metastatic tumors [5, 6]. The clinical response differs dramatically in different risk groups. The same variable pattern is observed regarding the five-year survival rates, where low- and intermediate-risk cases have survival rates over 90%, while high-risk patients have a survival rate of only 40-50%, despite the intensification of treatments and incorporation of immunotherapies. It is worth mentioning that high-risk NB is found in about 40% of cases and is often accompanied with chemoresistance and tumor relapse [5, 6]. The goal of current strategies for NB treatment is to decrease therapy- and minimize chemotherapy-related toxicity for patients from low and intermediate NB risk groups, whereas concerning high risk NB cases, present and upcoming trials are devoted to the development of novel immunotherapies, inhibitors of aberrant pathways (such as *MYC* and *ALK*), and radioisotope-containing regimens.

The current treatment of NB is based on stratification into low, medium, and high risk and includes observation, surgery, radiation therapy, ^131^I meta-iodobenzylguanidine therapy, chemotherapy, targeted therapy as well as high-dose chemotherapy, and radiation therapy with stem cell rescue [6, 7]. Various immunotherapy strategies are also discussed in the context of NB [8, 9].

In 2002, Daniels et al. [10] conducted a large study showing that breastfeeding was associated with a 41% (22–56%) reduction in risk of NB (odds ratio 0.6). Only two more studies with small *n* (*n* < 45) investigated the relationship between breastfeeding and NB and confirmed the conclusions of Daniels et al., which have been published [11, 12]. Numerous factors and agents affecting cancer cell proliferation are being investigated, but the mechanisms of inverse relationship between breastfeeding and NB still remain unclarified and the existence and importance of this relationship is overlooked.

Recent studies from our lab have shown the possible role of alpha-amylase (*α*-amylase), which is present in excessive amounts in breast milk, in the regulation of glucose metabolism [13–15]. Changes in glucose metabolism is recognized as being one of the hallmarks of cancer. Thus, *α*-amylase may have a role in the regulation of cell proliferation including that of cancer cells. There are some reports about antiproliferative effects of *α*-amylase *in vivo* with unknown mechanism [16–19].

Recently, we have shown that *α*-amylase inhibits cell proliferation and glucose uptake in human NB cell line SH-SY5Y [20]. Thus, the aim of our study was to confirm and extend our previous observations and determine if *α*-amylase treatment has any impact on the human NB cell lines SH-SY5Y, IMR-32, and LA-N-1, which partly correspond to the low-, medium-, and high-risk NB, respectively, and possess divergent genetic aberrations, by investigating effects on cell proliferation and glucose uptake. An investigation of the potential of the anticancer properties of *α*-amylase could result in the possibility to suggest a supplemental treatment with lower toxicity to current NB treatments, which is of undoubtful importance for the modern cure of this disease.

## 2. Materials and Methods

### 2.1. Cell Line Routine Culturing Conditions

The SH-SY5Y and IMR-32 cell lines were purchased from American Type Culture Collection (ATCC® CRL-2266™ and ATCC® CCL-127™, respectively, LGC Standards, Middlesex, UK), and LA-N-1 cell line was purchased from the European Collection of Authenticated Cell Cultures (ECACC 06041201, Salisbury, UK). The cell lines were tested for mycoplasma during the experimental period, and all were found to be negative (Eurofins Scientific, Cologne, Germany). Upon arrival, cell lines were thawed, amplified, and frozen in ampules in liquid nitrogen. Cells were seeded at a density range from 15000 cells/cm^2^ to 30000 cells/cm^2^ in tissue culture flasks (Nunc, Thermo Fisher Scientific, Roskilde, Denmark) containing 0.16 ml medium/cm^2^ and kept in a humidified incubator with 5% CO_2_ in air at 37°C. The cell lines were cultured in DMEM/Ham's F12 medium (Avantor VWR cooperation, Lund, Sweden) supplemented with 10% heat-inactivated fetal bovine serum (FBS) (Sigma-Aldrich Sweden AB, Stockholm, Sweden), 1 mM Na-pyruvate (Sigma-Aldrich Sweden AB), 1 mM nonessential amino acids (Avantor VWR cooperation), 100 *μ*g/ml streptomycin (Avantor VWR cooperation), 100 U/ml penicillin (Avantor VWR cooperation), 10 ng/ml epidermal growth factor (Lonza, Basel, Switzerland), and 2 mM L-glutamine (Avantor VWR cooperation). Cells were routinely passaged once a week. 0.05% trypsin/1 mM EDTA was used for cell detachment.

### 2.2. *α*-Amylase

Porcine *α*-amylase was purchased from Merck KGaA (catalogue number 10102814001, Darmstadt, Germany). The amylolytic activity in the solution was determined using ethylidene-pNP-G7 (4,6-ethylidenep-nitrophenyl-*α*, D-maltoheptaoside) as the substrate, according to the manufacturer's instructions (Infinity Amylase Liquid Stable Reagent; Thermo Fisher Scientific, Middletown, Virginia, USA), with modifications for a microtiter plate reader. The activity of the original amylase solution was found to be 1000 U/l. A dilution series of the *α*-amylase stock in 0.9% NaCl was made to be able to investigate the effect of *α*-amylase on the neuroblastoma cell lines SH-SY5Y, LA-N-1, and IMR-32.

### 2.3. Dose-Response Assay

**Figure 1:**
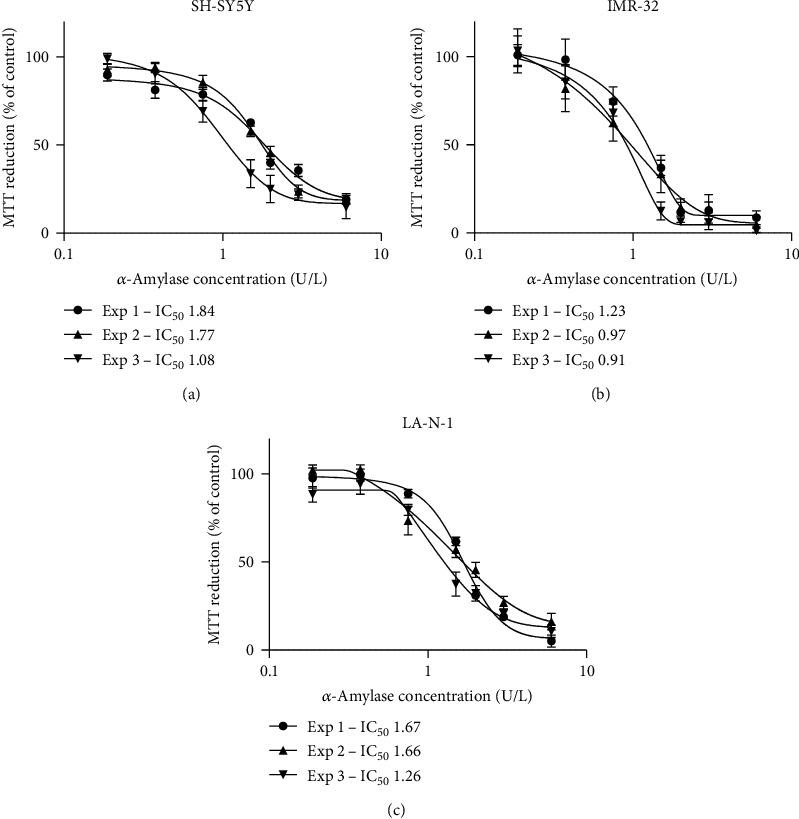
Treatment of NB cells with *α*-amylase decreases the cell number dose dependently. Human NB cells (a) SH-SY5Y, (b) IMR-32, and (c) LA-N-1 were incubated with porcine pancreatic *α*-amylase for 72 h, and then, an MTT assay was used for indirect determination of cell number. The IC_50_ of each repeat is presented on the right side of the graph. The results are expressed as percent of control. The data are derived from 6 wells per treatment from three different experiments and presented as mean ± SD.

Cells were detached, counted in a hemocytometer, and resuspended to a final concentration of 0.11 × 10^6^ cells/ml. A 180 *μ*l aliquot of the cell suspension was added per well in 96-well plates, and the plates were incubated for 24 h before addition of *α*-amylase. A dilution series of *α*-amylase was made in 0.9% NaCl, and 20 *μ*l aliquots were added to achieve the final concentrations shown in Figure 1. Controls received 20 *μ*l 0.9% NaCl. After 72 h of incubation, 20 *μ*l of 3-(4,5-dimethylthiazolyl-2)-2,5-diphenyltetrazolium bromide (MTT) (Sigma-Aldrich Stockholm AB) solution (5 mg/ml in phosphate-buffered saline) was added to each well, and the plates were returned to the CO_2_ incubator for 1 h. Thereafter, the MTT-containing medium was removed and the blue formazan product was dissolved by the addition of 100 *μ*l of 100% dimethyl sulfoxide per well. The plates were swirled gently for 15 min to dissolve the precipitate. The absorbance was measured at 540 nm using a Multiskan™ FC microplate spectrophotometer and the software SkanIt for Multiskan FC 3.1. Ink (Thermo Fisher Scientific). The results were analyzed using the GraphPad 9 software (La Jolla, California, USA). The presented IC_50_ values with 95% confidence interval are based on at least 3 dose-response experiments.

### 2.4. Digital Holographic Imaging and Analysis

To monitor the effect of *α*-amylase on motility, accumulated distance, and proliferation of neuroblastoma cells, the phase holographic microscope M4 (Phase Holographic Imaging AB, Lund, Sweden) was used. Cells were detached with 0.05% trypsin/1 mM EDTA and counted in a hemocytometer. SH-SY5Y cells were seeded at a density of 20000 cells/cm^2^, and LA-N-1 cells were seeded at a density of 30000 cells/cm^2^ in 6-well plates (Nunc, Thermo Fisher Scientific). After 24 h of incubation, the cell medium was aspirated, and 4 ml of fresh cell medium was added to each well. Subsequently, three wells were treated with 2 U/l *α*-amylase and the three remaining wells were treated with the equivalent volumes of 0.9% NaCl. After adding *α*-amylase, specific HoloLids™ (Phase Holographic Imaging AB) replaced the standard 6-well plate lid and the plate was placed on the stage of the M4 HoloMonitor inside a CO_2_ cell culturing incubator. Images were acquired every 5 minutes for 72 h using AppSuite™ (Phase Holographic Imaging AB). Following image acquisition, the time-lapses were used to analyze kinetic motility and proliferation of single cells and videos made using AppSuite™ (Phase Holographic Imaging AB).

### 2.5. Investigation of Glucose Uptake

For the detection of cellular glucose uptake, SH-SY5Y cells were seeded at a density of 20000 cells/cm^2^, and LA-N-1 and IMR-32 cells were seeded at a density of 30000 cells/cm^2^ in 12-well plates (Nalge Nunc International, Penfield, New York, USA) in 2 ml of regular growth medium and then incubated for 24 h. *α*-Amylase was administered 24 h after seeding as a sterile solution in 0.9% NaCl, to a final concentration of 2 U/l. Control received the same volume 0.9% NaCl. After 1 h of incubation without or with *α*-amylase, 2 *μ*l of ^3^H-radiolabelled deoxy-D-glucose (1 mCi/ml, catalogue number NET549A001MC, PerkinElmer, Boston, MA, USA) was added per well. The plates were incubated for 24 h. After incubation, cell lysates were prepared and mixed with 10 ml Ready Safe Liquid Scintillation Cocktail for Aqueous Samples (Beckman Coulter, Brea, CA, USA) in Poly-Q™ vials (Beckman Coulter). The vials were shaken for 2 min and stored for 48 h in the dark to reduce chemical quenching before analysis. ^3^H-glucose uptake was determined by liquid scintillation counting in a (Beckmann LS 6500 LSC multipurpose liquid scintillation counter (Beckmann Coulter)). All experiments were repeated at least three times.

### 2.6. Immunocytochemistry

Cells seeded at a density of 20000 cells/cm^2^ (SH-SY5Y) or 30000 cells/cm^2^ (LA-N-1 and IMR-32) in 6-well plates and treated with 2 U/L *α*-amylase for 72 h were used to investigate the effect of *α*-amylase on the expression of glucose transporter-1 (GLUT1) and glucose transporter-2 (GLUT2) receptors using immunocytochemistry. After 72 h of incubation, the cell medium was aspirated, and the cells were fixed with 3.7% formaldehyde in PBS (Merck KGaA). Subsequently, 400 *μ*l of blocking buffer (PBS with 1% bovine serum albumin (BSA)) was added to each well and the samples were incubated for a minimum of 2 h. After incubation, the blocking buffer was removed and 400 *μ*l recombinant Alexa Fluor® 488 Anti-Glucose Transporter GLUT1 antibody (1 : 400, ab195359, Abcam, Waltham, MA, USA) or recombinant Anti-Glucose Transporter GLUT2 antibody (1 : 400, ab234440, Abcam, Waltham, MA, USA) was added to separate wells and the samples incubated in the dark overnight. After washing, the secondary antibody Alexa Fluor 488™ anti-rabbit (1 : 800, A11034, Thermo Fisher Scientific) was added to the wells with GLUT2 unconjugated antibodies. Simultaneously, Alexa Flour™ 594 phalloidin (1 : 200, A12381, Thermo Fisher Scientific) was added to all wells. Both antibodies were diluted to a final volume of 400 *μ*l per well and were incubated for 1.5 h shielded from light. Finally, 400 *μ*l 4′,6-diamidino-2-phenylindole (DAPI) (1 *μ*g/ml, Thermo Fisher Scientific) was added to each well and the samples were incubated for 1.5 minutes in darkness. All wells were washed three times with PBS after each staining step, and all staining agents were diluted in blocking buffer. Finally, coverslips were mounted using Mowiol (Sigma-Aldrich Sweden AB). Samples were stored at 4°C and protected from light before microscopy. Confocal images were taken with a Leica SP8 DLS inverted confocal laser scanning microscope using an oil immersion 63x/1.4 objective lens (Leica Microsystems, Wetzlar, Germany).

### 2.7. Statistical Analyses

All data are presented as mean ± SD. A two-tailed Student's *t*-test with Holm-Sidak correction for multiple comparisons or two-way ANOVA with a Sidak correction for multiple comparisons was used to estimate differences. A Shapiro-Wilk test was used to assess the normality of distribution. In all statistical analyses, *p* ≤ 0.05 was considered significant. All analyses were carried out using Prism, version 9.3 (GraphPad Software, Inc., San Diego, CA, USA).

## 3. Results

### 3.1. Dose-Response Curves for NB Cells Treated with Alpha-Amylase

The antiproliferative effect of treating the human NB SH-SY5Y, IMR-32, and LA-N-1 cell lines with *α*-amylase was initially evaluated in dose-response experiments. Treatment with *α*-amylase at a final concentration above 0.2 U/l resulted in decreased MTT reduction, i.e., reduced cell number, with IC_50_ values 1.53 ± 0.23 U/l for SH-SY5Y, 1.04 ± 0.17 for IMR-32, and 1.56 ± 0.42 for LA-N-1 cell lines after 72 h of incubation (Figure 1). These data gave us the basis for the treatment concentration of *α*-amylase in further studies.

### 3.2. Cell Proliferation

**Figure 2:**
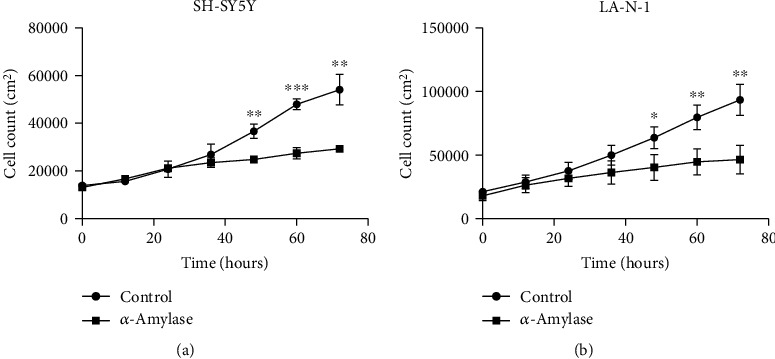
*α*-Amylase treatment inhibits the proliferation of (a) SH-SY5Y and (b) LA-N-1 cells. *α*-Amylase was added to the final concentration of 2 U/l (control received 0.9% NaCl) at 24 h after seeding. The cell number was determined by digital holographic microscopy. The curves are drawn from cell count data obtained at the indicated times after start of the time-lapse. The data are derived from three images per well and 6 wells per treatment from two different experiments. Data are represented as mean ± SD. ∗ indicates a statistical significance between *α*-amylase treated and control in the corresponding time points when *p* < 0.05 and ∗∗ when *p* < 0.01.

Growth curves for NB cells treated with 2 U/l *α*-amylase were established by digital holography. *α*-Amylase treatment significantly reduced number of SH-SY5Y cells by 32% (*p* = 0.0125) and LA-N-1 cells by 37% (*p* = 0.0036), already after 48 h of incubation (Figures 2(a) and 2(b), respectively). This decline in cell count was observed until the end of the time-lapse and reached 46% (*p* = 0.0026) and 50% (*p* = 0.014) for SH-SY5Y and LA-N-1 cells, respectively (Figure 2).

### 3.3. Treatment with Pancreatic *α*-Amylase Inhibits Cell Movement in SH-SY5Y and LA-N-1 Cell Lines

**Figure 3:**
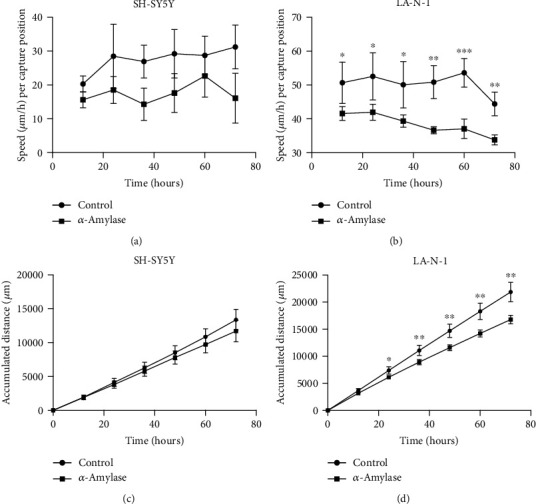
The motility and accumulated distance of (a, c) SH-SY5Y and (b, d) LA-N-1 cells treated with *α*-amylase. *α*-Amylase was added to the final concentration of 2 U/l (control received 0.9% NaCl) at 24 h after seeding. The data are derived from three images per well and 6 wells per treatment from two different experiments. Data are represented as mean ± SD. ∗ indicates a statistical significance between *α*-amylase treated and control in the corresponding time points when *p* < 0.05 and ∗∗ when *p* < 0.01.

Cell motility and accumulated distance data from control and amylase-treated wells collected every 5 minutes after start of the time-lapse were compared using Student's paired *t*-test. Motility and accumulated distance were not significantly different for *α*-amylase-treated SH-SY5Y cells (Figures 3(a) and 3(c), respectively) when compared to control. However, *α*-amylase treatment significantly reduced the motility and accumulated distance of LA-N-1 cell line, where motility (Figure 3(b)) was reduced by 18% (*p* = 0.0304) at 12 h of treatment and this decline reached 24% (*p* = 0.014) at 72 h of treatment which is reflected in the reduced accumulated distance in *α*-amylase-treated cells compared to control (Figure 3(d)).

### 3.4. Treatment with *α*-Amylase Inhibits ^3^H-Glucose Uptake

**Figure 4:**
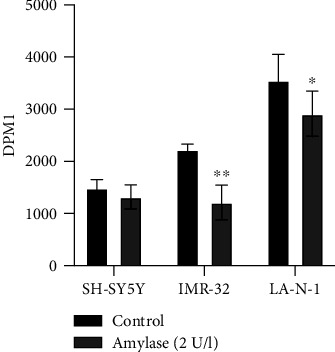
^3^H-glucose uptake in SH-SY5Y, IMR-32, and LA-N-1 cells treated with *α*-amylase. *α*-Amylase was added to the final concentration of 2 U/l (control received 0.9% NaCl) at 24 h after seeding. One hour later, ^3^H-glucose was added to the medium. After 24 h of incubation in the presence of ^3^H-glucose, the uptake was determined by liquid scintillation counting. Data are represented as mean of six independent samples ± SD. ∗ indicates a statistical significance between *α*-amylase treated and control when *p* < 0.05 and ∗∗ when *p* < 0.01.

Addition of *α*-amylase to the medium (2 U/l) significantly decreased the uptake of ^3^H-glucose in IMR-32 and LA-N-1 cells after 24 h of incubation (Figure 4). The glucose uptake in *α*-amylase-treated IMR-32 cells at 24 h of treatment was 55% of control values (*p* = 0.0020) and 82% of control in LA-N-1 cells (*p* = 0.0027) (Figure 4). Although there was a trend to decreased glucose uptake in SH-SY5Y cells treated with *α*-amylase for 24 h, it was not significant.

### 3.5. Glucose Transporter Expression

**Figure 5:**
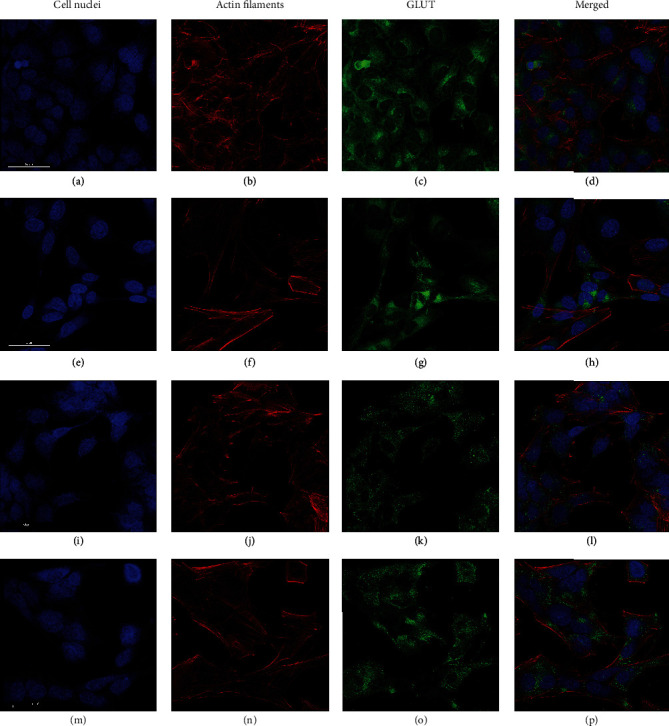
Images of the expression of GLUT1 and GLUT2 in control SH-SY5Y cells and cells treated with *α*-amylase for 72 h. *α*-Amylase was added to the final concentration of 2 U/l (control received 0.9% NaCl) at 24 h after seeding. GLUT1: (a–d) control; (e–h) *α*-amylase. GLUT2: (i–l) control; (m–p) *α*-amylase. NB cells were fixed in 3.7% formaldehyde and stained to visualize cell nuclei (DAPI, blue), actin filaments (Alexa Flour™ 594, red), and GLUT1 or GLUT2 (Alexa Flour™ 488, green). The scale bar is 50 *μ*m.

**Figure 6:**
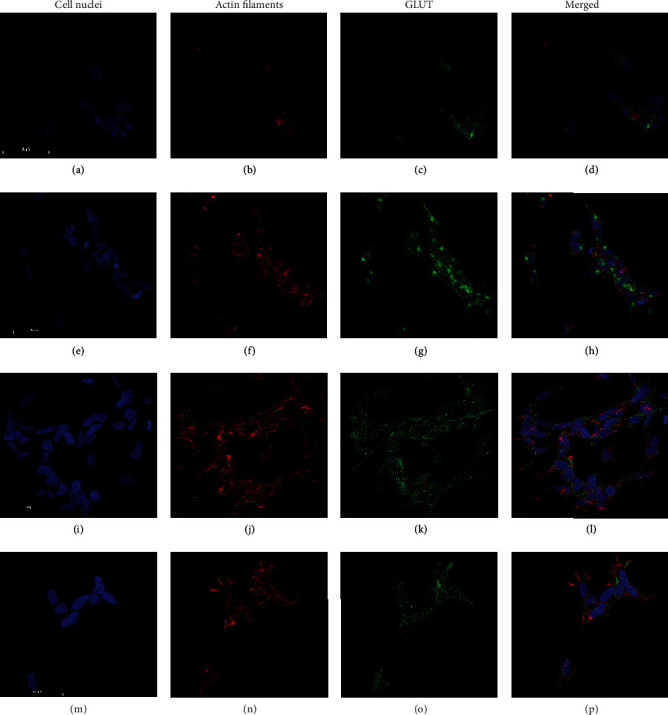
Images of the expression of GLUT1 and GLUT2 in IMR-32 cells treated with *α*-amylase for 72 h. *α*-Amylase was added to the final concentration of 2 U/l (control received 0.9% NaCl) at 24 h after seeding. GLUT1: (a–d) control; (e–h) 2 U/l *α*-amylase. GLUT2: (i–l) control; (m–p) 2 U/l *α*-amylase. NB cells were fixed in 3.7% formaldehyde and stained to visualize cell nuclei (DAPI, blue), actin filaments (Alexa Flour™ 594, red), and GLUT1 or GLUT2 (Alexa Flour™ 488, green). The scale bar is 50 *μ*m.

**Figure 7:**
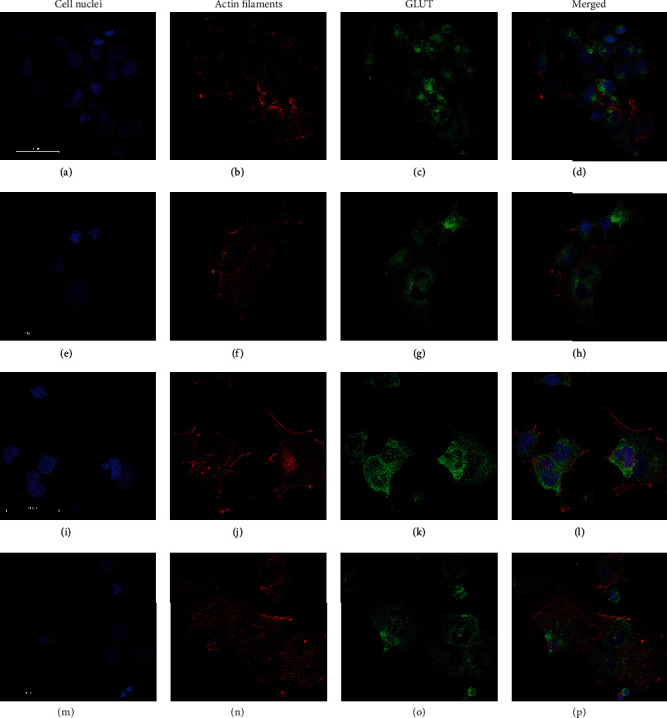
Images of the expression of GLUT1 and GLUT2 in LA-N-1 cells treated with *α*-amylase for 72 h. *α*-Amylase was added to the final concentration of 2 U/l (control received 0.9% NaCl) at 24 h after seeding. GLUT1: (a–d) control; (e–h) 2 U/l *α*-amylase. GLUT2: (i–l) control; (m–p) 2 U/l *α*-amylase. NB cells were fixed in 3.7% formaldehyde and stained to visualize cell nuclei (DAPI, blue), actin filaments (Alexa Flour™ 594, red), and GLUT1 or GLUT2 (Alexa Flour™ 488, green). The scale bar is 50 *μ*m.

Immunocytochemistry was performed to analyze the expression of GLUT1 and GLUT2 in SH-SY5Y (Figure 5), IMR-32 (Figure 6), and LA-N-1 cells (Figure 7) and also if *α*-amylase treatment affected the expression. Expression of both GLUT1 and GLUT2 was clearly demonstrated for all cell lines (Figures 5–7) in both control and *α*-amylase-treated cultures. Visual inspection does not reveal any differences in expression, and semiquantitative evaluation of expression was not performed.

## 4. Discussion

In this pilot study, the effect of treating human NB cells with pancreatic *α*-amylase was investigated. Pancreatic amylase was studied as a potential anticancer agent in the beginning of the 20th century [16–18] for the treatment of tumors at different locations in the body. Then, after almost a century of silence, interest in this enzyme in the context of cancer was raised again as Novak and Trnka reported improved survival of mice treated with amylase after transplantation of melanoma cells [19]. More recently, salivary *α*-amylase was shown to have antiproliferative effects in primary cell cultures of rat mammary epithelial cells and human breast cancer cells [21]. Moreover, breast cancer survivors have an elevated blood level of salivary *α*-amylase [22], which has been recognized as a stress marker. However, it is unknown if the elevated salivary *α*-amylase activity is the consequence of cancer and stress or if it is an attempt of the organism to cure itself.

As previously mentioned, numerous factors and agents affecting NB are being investigated, but the mechanisms of the inverse relationship between breastfeeding and NB described by Daniels et al. [10] still remain unclarified and the existence and importance of this relationship are overlooked. An internalization and transcytosis of pancreatic amylase by enterocytes resulting in increased serum amylase level were shown by Cloutier et al. [23]. Thus, it is possible that breast milk *α*-amylase may increase the blood level of *α*-amylase in breastfed infants.

We initiated our study with dose-response curve experiments where the dose range was chosen based on serum amylase in humans. The antiproliferative effect was observed at concentrations below the normal human serum range of *α*-amylase (40-120 U/l), which is comparable to the low level of *α*-amylase found in infant serum during the first eight months after birth [24, 25]. We found IC_50_ values of 1.53 ± 0.23 U/l for SH-SY5Y, 1.04 ± 0.17 for IMR-32, and 1.56 ± 0.42 for LA-N-1, thus showing that observed effects could be reached by small doses within the physiological range of serum amylase activity in infants. A possible explanation for the variation in results is that the neuroblastoma cell lines contain two cellular phenotypic variants of undifferentiated mesenchymal cells and committed adrenergic cells [26]. The cell types have divergent gene expression profiles and are epigenetically regulated which allows for interconversion between the two phenotypes [27]. Additionally, the cell lines possess different genetic aberrations. The LA-N-1 cell line carries genetic aberrations such as a mutated *p53* gene, *MYCN* amplification, and silenced caspase 8 expression [28–30]. The SH-SY5Y and IMR-32 cell lines carry wild-type *p53* combined with a silenced caspase 8 expression [29, 30]. The IMR-32 cell line also carries an amplification of the *MYCN* gene, and the SH-SY5Y cell line has trisomy 1q [31, 32]. Similar variation in *α*-amylase sensitivity among cell lines has also been observed in breast cancer cell lines [21]. Despite the diverse genetic abnormalities and cellular phenotypic variants in the cell lines, *α*-amylase treatment had an antiproliferative effect on all three cell lines. The levels of *α*-amylase activity observed in breast milk (about 9-10 kU/l) and in the postprandial duodenal aspirates of infants (about 5 kU/l) are high considering the absence of starch in the infants' diet [33–37], but they are significantly lower when compared to those found in the duodenal lumen of healthy adults [38]. One should remember that preprandial duodenal fluid samples in healthy term newborns do not contain any amylase at least during the first month of life [39], and in fact, all healthy term newborns which are not breastfed are exocrine pancreatic insufficient [40]. Thus, the mothers' milk seems to be an important source of *α*-amylase, which can enter the circulation and may affect NB development or have some other yet unknown functions unrelated to carbohydrate digestion.

The inhibition of cell proliferation by *α*-amylase treatment of SH-SY5Y and LA-N-1 cells was also proven by time-lapse imaging using digital holographic microscopy (significance found after 48 h of treatment). Interestingly, the digital holographic microscopy revealed a significant effect on cell motility and accumulated distance of LA-N-1 cells, but not SH-SY5Y cells, already after 24 h of *α*-amylase treatment. Unfortunately, IMR-32 cell line is not suitable for digital holographic microscopy due to growth peculiarities such as tight cell crowding; thus, data on cell growth, motility, and migration for this particular line was not obtained.

As a recent study from our laboratory shows the possible role of *α*-amylase in the regulation of glucose metabolism [13–15] and it is well known that changes in glucose metabolism are recognized as being one of the hallmarks of cancer, the effect of *α*-amylase on glucose uptake in SH-SY5Y, IMR-32, and LA-N-1 cells was investigated. Here, we show that the cellular glucose uptake in IMR-32 and LA-N-1 cells was significantly inhibited already after 24 h of incubation with *α*-amylase while there was only a trend for inhibition in SH-SY5Y cells. We have seen that ^3^H-glucose uptake was decreased in SH-SY5Y after 72 h of treatment [20]. This decreased uptake of glucose may be the cause for the inhibition of cell proliferation and cell motility.

Glucose transporter inhibitors are well-recognized targets for anticancer therapy [41, 42]. Some novel anticancer agents, such as the *α*-tocopherol derivative ESeroS-G, show the ability to target energy metabolism of cancer cells and inhibit their migration [43]. Recently, *α*-amylase was shown to actively participate in the regulation of cell proliferation and turnover in the duodenum [44], and the regulatory abilities of this molecule in the intestine are obvious. It is known that SH-SY5Y cells express both GLUT1 and GLUT2 transporters. Previously, we have shown *α*-amylase-dependent changes in insulin secretion in pancreatic *β* cells, which express GLUT2 [15], thus supporting our notion that at least glucose uptake by GLUT2 may be affected by *α*-amylase in neuroblastoma cells. *α*-Amylase's inhibitory effect on glucose uptake could be exerted in various ways such as blocking the glucose transporters or affecting the expression of the transporters. Thus, we have chosen immunocytochemistry as an initial step to evaluate GLUT1 and GLUT2 expression in the cell lines and to investigate if *α*-amylase treatment influenced the expression level. The preliminary data do not reveal any difference in GLUT1 and GLUT2 expression in SH-SY5Y, IMR-32, and LA-N-1 cells and there appeared to be no effect of *α*-amylase treatment. However, only the qualitative assessment was performed and the data obtained should be supported by quantitative investigations. Nevertheless, the demonstrated expression of both GLUT1 and GLUT2 in all NB cell lines used in the study is an important finding which highlights the mechanisms of glucose transport in this type of tumor.

Some limitations of our studies include the usage of porcine pancreatic *α*-amylase instead of human salivary *α*-amylase which is present in breast milk, which might lead to a discrepancy in results [33]. However, salivary *α*-amylase and pancreatic *α*-amylase share 97% homology and both isoenzymes can diffuse into the bloodstream, which supports the use of pancreatic *α*-amylase [45]. Human salivary *α*-amylase treatment at similar concentrations has previously been studied in breast cancer cells [21]. Additionally, the effect of *α*-amylase treatment is not evaluated on a healthy human cell line as a control. The detailed investigation of changes in glucose transporter expression after amylase treatment should be performed as well. However, despite the mentioned limitations, the current work demonstrates the novel important concept of *α*-amylase as a homeostatic agent and an important player in the metabolic regulations throughout the whole body during the entire lifespan.

## 5. Conclusions

The described study was designed as a proof-of-concept study, and the mechanisms underlying the observed effects remain to be thoroughly elucidated. However, the ability of *α*-amylase to inhibit cell proliferation and cell motility in human NB cells is obvious and this enzyme should be investigated as a possible mild, nontoxic alternative or/and supplementation to the current NB treatment strategies.

## Data Availability

All data relevant to the study are included in the article.

## References

[1] Davis S., Rogers M. A., Pendergrass T. W. (1987). The incidence and epidemiologic characteristics of neuroblastoma in the United States. *American Journal of Epidemiology*.

[2] Gurney J. G., Ross J. A., Wall D. A., Bleyer W. A., Severson R. K., Robison L. L. (1997). Infant cancer in the US: histology-specific incidence and trends, 1973 to 1992. *Journal of Pediatric Hematology/Oncology*.

[3] Brodeur G. M., Marris J. M., Pizzo P. A., Poplack B. G. (2006). Neuroblastoma. *Principles and Practice of Pediatric Oncology*.

[4] Maris J. M., Hogarty M. D., Bagatell R., Cohn S. L. (2007). Neuroblastoma. *Lancet*.

[5] Yamamoto K., Ohta S., Ito E. (2002). Marginal decrease in mortality and marked increase in incidence as a result of neuroblastoma screening at 6 months of age: cohort study in seven prefectures in Japan. *Journal of Clinical Oncology*.

[6] Hiyama E., Iehara T., Sugimoto T. (2008). Effectiveness of screening for neuroblastoma at 6 months of age: a retrospective population-based cohort study. *Lancet*.

[7] Irwin M. S., Park J. R. (2015). Neuroblastoma: paradigm for precision medicine. *Pediatric Clinics of North America*.

[8] Boes M. (2018). Cancer immunotherapy: moving beyond checkpoint inhibition. *Oncotarget*.

[9] Ishfaq M., Pham T., Beaman C., Tamayo P., Yu A. L., Joshi S. (2021). BTK inhibition reverses MDSC-mediated immunosuppression and enhances response to anti-PDL1 therapy in neuroblastoma. *Cancers*.

[10] Daniels J. L., Olshan A. F., Pollock B. H., Shah N. R., Stram D. O. (2002). Breast-feeding and neuroblastoma, USA and Canada. *Cancer causes & control: CCC*.

[11] Smulevich V. B., Solionova L. G., Belyakova S. V. (1999). Parental occupation and other factors and cancer risk in children: I. Study methodology and non-occupational factors. *International Journal of Cancer*.

[12] Hardell L., Dreifaldt A. C. (2001). Breast-feeding duration and the risk of malignant diseases in childhood in Sweden. *European Journal of Clinical Nutrition*.

[13] Pierzynowski S. G., Gregory P. C., Filip R., Woliński J., Pierzynowska K. (2018). Glucose homeostasis dependency on acini-islet-acinar (AIA) axis communication: a new possible pathophysiological hypothesis regarding diabetes mellitus. *Nutrition & Diabetes*.

[14] Pierzynowska K. G., Lozinska L., Woliński J., Pierzynowski S. (2018). The inverse relationship between blood amylase and insulin levels in pigs during development, bariatric surgery, and intravenous infusion of amylase. *PLoS One*.

[15] Pierzynowska K., Oredsson S., Pierzynowski S. (2020). Amylase-dependent regulation of glucose metabolism and insulin/glucagon secretion in the streptozotocin-induced diabetic pig model and in a rat pancreatic beta-cell line, BRIN-BD11. *Journal Diabetes Research*.

[16] Wiggin F. H. (1906). Case of multiple fibrosarcoma of the tongue, with remarks on the use of trypsin and amylopsin in the treatment of malignant disease. *Journal of the American Medical Association*.

[17] Goeth R. A. (1907). Pancreatic treatment of cancer, with report of a cure. *JAMA*.

[18] Little W. L. (1908). A case of malignant tumor, with treatment. *Journal of the American Medical Association*.

[19] Novak J., Trnka F. (2005). Proenzyme therapy of cancer. *Anticancer Research*.

[20] Pierzynowska K., Oredsson S. (2022). ESPGHAN 54th Annual Meeting Abstracts. *Pediatric Gastroenterology and Nutrition*.

[21] Fedrowitz M., Hass R., Bertram C., Löscher W. (2011). Salivary *α*-amylase exhibits antiproliferative effects in primary cell cultures of rat mammary epithelial cells and human breast cancer cells. *Journal of Experimental & Clinical Cancer Research*.

[22] Wan C., Couture-Lalande M. È., Narain T. A., Lebel S., Bielajew C. (2016). Salivary alpha-amylase reactivity in breast cancer survivors. *International Journal of Environmental Research and Public Health*.

[23] Cloutier M., Gingras D., Bendayan M. (2006). Internalization and transcytosis of pancreatic enzymes by the intestinal mucosa. *The Journal of Histochemistry and Cytochemistry*.

[24] Nakajima K., Nemoto T., Muneyuki T., Kakei M., Fuchigami H., Munakata H. (2011). Low serum amylase in association with metabolic syndrome and diabetes: a community-based study. *Cardiovascular Diabetology*.

[25] Otsuki M., Yuu H., Saeki S., Baba S. (1977). The characteristics of amylase activity and the isoamylase pattern in serum and urine of infants and children. *European Journal of Pediatrics*.

[26] van Groningen T., Koster J., Valentijn L. J. (2017). Neuroblastoma is composed of two super-enhancer-associated differentiation states. *Nature Genetics*.

[27] van Groningen T., Akogul N., Westerhout E. M. (2019). A NOTCH feed-forward loop drives reprogramming from adrenergic to mesenchymal state in neuroblastoma. *Nature Communications*.

[28] Hopkins-Donaldson S., Bodmer J. L., Bourloud K. B., Brognara C. B., Tschopp J., Gross N. (2000). Loss of caspase-8 expression in highly malignant human neuroblastoma cells correlates with resistance to tumor necrosis factor-related apoptosis-inducing ligand-induced apoptosis. *Cancer Research*.

[29] Davidoff A. M., Pence J. C., Shorter N. A., Iglehart J. D., Marks J. R. (1992). Expression of p53 in human neuroblastoma- and neuroepithelioma-derived cell lines. *Oncogene*.

[30] Huang R., Cheung N.-K. V., Vider J. (2011). MYCN and MYC regulate tumor proliferation and tumorigenesis directly through BMI1 in human neuroblastomas. *The FASEB Journal*.

[31] Harenza J. L., Diamond M. A., Adams R. N. (2017). Transcriptomic profiling of 39 commonly-used neuroblastoma cell lines. *Scientific Data*.

[32] Neuroblastoma T. C., Lines C. (2002). Neuroblastoma. *Journal Human Cell Culture*.

[33] Lindberg T., Skude G. (1982). Amylase in human milk. *Pediatrics*.

[34] Hegardt P., Lindberg T., Börjesson J., Skude G. (1984). Amylase in human milk from mothers of preterm and term infants. *Journal of Pediatric Gastroenterology and Nutrition*.

[35] Dewit O., Dibba B., Prentice A. (1990). Breast-milk amylase activity in English and Gambian mothers: effects of prolonged lactation, maternal parity, and individual variations. *Pediatric Research*.

[36] Pierzynowski S. G., Weström B. R., Svendsen J., Svendsen L., Karlsson B. W. (1995). Development and regulation of porcine pancreatic function. *International Journal of Pancreatology*.

[37] Heitlinger L. A., Lee P. C., Dillon W. P., Lebenthal E. (1983). Mammary amylase: a possible alternate pathway of carbohydrate digestion in infancy. *Pediatric Research*.

[38] Worning H., Müllertz S., Thaysen E. H., Bang H. O. (1967). pH and concentration of pancreatic enzymes in aspirates from the human duodenum during digestion of a standard meal. In patients with duodenal ulcer and in patients subjected to different gastric resections. *Scandinavian Journal of Gastroenterology*.

[39] Lebenthal E., Lee P. C. (1980). Development of functional responses in human exocrine pancreas. *Pediatrics*.

[40] Struyvenberg M. R., Martin C. R., Freedman S. D. (2017). Practical guide to exocrine pancreatic insufficiency - breaking the myths. *BMC Medicine*.

[41] Peng Y., Xing S. N., Tang H. Y. (2019). Influence of glucose transporter 1 activity inhibition on neuroblastoma in vitro. *Gene*.

[42] Reckzeh E. S., Waldmann H. (2020). Small-molecule inhibition of glucose transporters GLUT-1–4. *Chembiochem*.

[43] Zhao L., Zhao X., Zhao K. (2014). The *α*-tocopherol derivative ESeroS-GS induces cell death and inhibits cell motility of breast cancer cells through the regulation of energy metabolism. *European Journal of Pharmacology*.

[44] Date K., Yamazaki T., Toyoda Y., Hoshi K., Ogawa H. (2020). *α*-Amylase expressed in human small intestinal epithelial cells is essential for cell proliferation and differentiation. *Journal of Cellular Biochemistry*.

[45] Azzopardi E., Lloyd C., Teixeira S. R., Conlan R. S., Whitaker I. S. (2016). Clinical applications of amylase: novel perspectives. *Surgery*.

